# The Book World of Medicine and Science

**Published:** 1898-08-06

**Authors:** 


### The Book World of Medicine and Science.

Sir Benjamin Collins Brodie. By Timothy Holmes, M.A., F.R.C.S. (London: T. Fisher Unwin. 1898. Price 3s. 6d.)

Undoubtedly Sir Benjamin Brodie was one of the masters of medicine; but he was something else, and the position he held in society for a long series of years renders his life something much more than a mere medical biography. The interest of this particular record of the life of Brodie does not, however, depend upon the fact that the subject of the memoir was a many-sided man, with broad sympathies, who was in touch with all that was going on, and was more or less intimate with the best people of his day. No doubt such a life would furnish good matter to any literary man out of which to weave a readable biography; but the interest of this book goes much further, and hinges largely on the fact that it is written by one who has a thorough knowledge of the setting in which the life was lived, who, in dealing with matters which concern St. George's Hospital, is on his own ground, and in describing the reforms with which Sir Benjamin Brodie was concerned is treating of matters with which he himself has also been interested for years, and upon which he is no mean authority. At the present day, when under the influence of reaction many men are ready to assert that a return to some kind of substitute for the old apprenticeship system is necessary for the complete education of our medical students, it is curious and not devoid of importance to find that Brodie attributed the low education and tastes of the medical students of his day to "the absurd system of apprenticeship to an apothecary, which custom formerly, and since that an Act of Parliament, has imposed upon on what are called general practitioners." In fact, he appeared to look upon apprenticeship as a barrier by which men of culture were kept out of the profession. Among the great changes which have taken place during this century in hospital management is one of which we are reminded in this memoir, for at the time when Brodie was appointed assistant surgeon at St. George's Hospital that position was a very different one from what it is at the present day. The fact is that in those days, when there were no out-patients except those who were completing the cure which had been commenced in the wards, the old assistants, instead of being mainly occupied, as is now the case, in seeing out-patients, were appointed to assist individual members of the staff in the care of their in-patients. Hence Brodie was appointed as Home's assistant, the first real assistant surgeon to St. George's Hospital, in the modern sense, not having been appointed until the year 1829. "So comparatively recent is the growth of that out patient system which now lies like an incubus on all our London hospitals." As a highly-cultured man, and as one who was able to hold his own among the leaders of thought in scientific and literary circles, Sir Benjamin Brodie well deserved his success, and the same many-sided activity which was really the secret of the position which he held during his life undoubtedly adds greatly to the interest of his biography, which, as it comes from the hands of Mr. Holmes, will be found pleasant and suggestive reading, not only by members of our own profession, but by that large circle of educated people whose interest in such a work is likely to be chiefly historical and literary.

Rheumatoid Arthritis. By Gilbert A Bannatyne, M.D.Glas., M.R.C.P.E., Hon. Physician to the Royal United Hospital and to the Royal Mineral Water Hospital, Bath. (Bristol: 1898. John Wright and Co. Second edition, pp. 182. With plates and illustrations. 7s. 6d. nett.)

Rheumatoid arthritis is one of the most puzzling subjects in the whole domain of medicine; its recognition has only recently come about, its pathology is obscure, and its very name is unknown on the Continent and not universally accepted here. The difficulty of differentiating it from other chronic joint affections may be extreme, and its treatment is even more unsatisfactory than its pathology. We have, therefore, every reason to be grateful to Dr. Bannatyne for this second edition of an honest attempt to surmount the obstacles associated with this disease. The key to the author's views may be found in the discovery made by Dr. Wohlmann and himself, and confirmed by Dr. Blaxall, of a bacillus, believed to be specific, in the synovial fluid and membrane and blood of acute cases. It is admitted that inoculation experiments have so far failed, but Dr. Bannatyne states that the bacillus is certainly present in this disease and in no other; he says candidly that its actual relation to the pathology of rheumatoid arthiritis cannot as yet be definitely stated, but at the same time he is free to believe that it is the actual materies morbi. He consequently considers the joint symptoms to be primary, indicating the initial lesions, while the nervous symptoms are secondary. In other words, he holds that the disease results from the growth of a micro organism in the joints, and that this brews toxic products which act upon the central nervous system, producing, among other effects, the skin changes and the muscular atrophy. Turning to the pathological anatomy, Dr. Bannatyne describes this with care and accuracy; his illustrations of the histology are, however, rather crude. It is to be noted that, contrary to what is perhaps the most general view, he considers that the first changes take place in the synovial membrane, the cartilage being affected later. The author recognises two forms of rheumatoid arthritis—acute and chronic—and from both of these he distinguishes senile arthritis of the hip and shoulder. This was formerly described as the centrifugal form of rheumatoid arthritis; but Dr. Bannatyne regards it as a totally different disease of a degenerative character. The account of the symptoms is very good, being based on an analysis of 293 personally observed cases; the points in diagnosis are also well given. With reference to treatment a liberal diet is advised, and a large number of drugs having been passed under review guaiacol carbonate is recommended as the most satisfactory. Thermal and electrical treatment are also advocated, and the waters of Bath not unnaturally receive high praise. It should be noted, however, that all chronic cases which can afford it are recommended to winter in Egypt. The book gives evidence of careful study and wide reading; the bibliographical references are unusually copious and the general arrangement is lucid; the plates are excellent, but the woodcuts rather inferior.

